# Attentional Modulation of Eye Blinking Is Altered by Sex, Age, and Task Structure

**DOI:** 10.1523/ENEURO.0296-23.2024

**Published:** 2024-03-01

**Authors:** Isabell C. Pitigoi, Brian C. Coe, Olivia G. Calancie, Donald C. Brien, Rachel Yep, Heidi C. Riek, Ryan H. Kirkpatrick, Blake K. Noyes, Brian J. White, Gunnar Blohm, Douglas P. Munoz

**Affiliations:** Centre for Neuroscience Studies, Queen’s University, Kingston, Ontario K7L 3N6, Canada

**Keywords:** anti-saccade, blink timing, free-viewing, inhibitory control, visual processing

## Abstract

Spontaneous eye blinking is gaining popularity as a proxy for higher cognitive functions, as it is readily modulated by both environmental demands and internal processes. Prior studies were impoverished in sample size, sex representation, and age distribution, making it difficult to establish a complete picture of the behavior. Here we present eye-tracking data from a large cohort of normative participants (*n *= 604; 393 F; aged 5–93 years) performing two tasks: one with structured, discrete trials (interleaved pro-/anti-saccade task, IPAST) and one with a less structured, continuous organization in which participants watch movies (free-viewing; FV). Sex- and age-based analyses revealed that females had higher blink rates between the ages of 22 and 58 years in the IPAST and 22 and 34 years in FV. We derived a continuous measure of blink probability to reveal behavioral changes driven by stimulus appearance in both paradigms. In the IPAST, blinks were suppressed near stimulus appearance, particularly on correct anti-saccade trials, which we attribute to the stronger inhibitory control required for anti-saccades compared with pro-saccades. In FV, blink suppression occurred immediately after scene changes, and the effect was sustained on scenes where gaze clustered among participants (indicating engagement of attention). Females were more likely than males to blink during appearance of novel stimuli in both tasks, but only within the age bin of 18–44 years. The consistency of blink patterns in each paradigm endorses blinking as a sensitive index for changes in visual processing and attention, while sex and age differences drive interindividual variability.

## Significance Statement

Eye-tracking is becoming useful as a noninvasive tool for detecting preclinical markers of neurological and psychiatric disease. Blinks are understudied despite being an important supplement to saccade and pupil eye-tracking metrics. The present study is a crucial step in developing a healthy baseline for blink behavior to compare to clinical groups. While many prior blink studies suffered from small sample sizes with relatively low age and sex diversity (review by Jongkees and Colzato, 2016), our large cohort of healthy participants has permitted a more detailed analysis of sex and age effects in blink behavior. Furthermore, our analysis techniques are robust to temporal changes in blink probability, greatly clarifying the relationship between blinking, visual processing, and inhibitory control mechanisms on visual tasks.

## Introduction

Spontaneous eye blinks are those which occur periodically, without voluntary control or sensory stimulation, to replenish corneal tear film to maintain clear vision. These blinks produce visual blackouts ∼10–20 times a minute, lasting ∼300 ms each, which amounts to nearly 10% of the time spent awake (Doughty, 2001; Casse et al., 2007; Kwon et al., 2013). Consequently, to maintain visual efficiency, blinks are strategically timed to limit co-occurrence with important visual or auditory events (Shultz et al., 2011; Hoppe et al., 2018). This implies integration of bottom-up sensory signals and top-down conscious goals to prioritize efficient information gathering (Murali and Händel, 2021). Spontaneous blinking is thus modulated by both internal and external factors including attention (Siegle et al., 2008; Nakano et al., 2009; Wascher et al., 2015; Maffei and Angrilli, 2018, 2019), cognitive control (van Bochove et al., 2012), and even social communication (Hömke et al., 2018). Interindividual variability in blink rates is very high, driven by physiological and psychological factors (Doughty and Naase, 2006; Hoppe et al., 2018; van Slooten et al., 2019). Therefore, the primary goals of this study are to characterize (1) the full range of blink behavior within healthy individuals using sex and age as factors and (2) the unique strategies for blink timing on tasks with different structures and cognitive demands.

Sex differences in blink behavior are disputed; higher blink rates have been observed in females than in males (Bentivoglio et al., 1997; Sforza et al., 2008; Coors et al., 2021), while others identified the opposite (Dalcegio et al., 2012) or no effect of sex (Doughty, 2002; Buitenweg et al., 2021; Calancie et al., 2022; Mbamba et al., 2023). Age-related differences have not been consistently found either (Bentivoglio et al., 1997; Chen et al., 2003; Doughty and Naase, 2006; Sforza et al., 2008; Coors et al., 2021; Mbamba et al., 2023). As previously suggested (Jongkees and Colzato, 2016; Unsworth et al., 2019), it is likely that prior studies were underpowered to detect an effect due to small sample sizes or limited analytical techniques. To fill this gap, we analyzed blink data from 604 participants (393 F), aged 5–93 years inclusive, to explore sex and age effects more robustly.

The precise role of blinking in visual processing has been notoriously difficult to characterize due the sensitivity of blinking to task design. Task structure, modality, and complexity all influence the timing and frequency of blinks, complicating our understanding of underlying processes (review by Stern et al., 1984). In tasks with discrete trial structure, blinks mark the start and end of a period of stimulus processing and/or response execution (Siegle et al., 2008; Wascher et al., 2015; Calancie et al., 2022, 2023). Here we used an interleaved pro-/anti-saccade task (IPAST) to assess blink timing within a discretely structured task with low perceptual load and varying cognitive difficulty. Anti-saccade trials are difficult to perform correctly as they require inhibitory control (Munoz and Everling, 2004). We therefore expect anti-saccade trials to coincide with lower blink probability, as spontaneous blinking is believed to index inhibitory control (Colzato et al., 2009).

To contrast the IPAST, we presented participants with a less structured video free-viewing (FV) task. This was a dynamic visual stream producing a high perceptual load yet requiring little to no cognitive effort (Tseng et al., 2013). In such tasks with continuous trials, blinks tend to occur when information intake requirements are momentarily reduced, for example, during movie scenes where the main character is absent or actions have concluded (Nakano et al., 2009), rather than during interesting or task-relevant ones (Nakano and Miyazaki, 2019; Ranti et al., 2020). In FV, we expect blinking to be suppressed at the start of a new clip and remain low on clips with high gaze clustering between participants, signalling engagement of attention. Further, we expect FV to produce lower blink rates and durations due to the continuous perceptual load compared with IPAST which has predictable breaks.

## Materials and Methods

### Participants

Experimental procedures were reviewed for ethical compliance by the Queen's University Faculty of Health Sciences and Affiliated Teaching Hospitals Research Ethics Board. We recruited healthy males and females aged 5 and older from the greater area of Kingston, Ontario, Canada, through online and newspaper advertisements. All participants had normal or corrected-to-normal vision and reported no history of neurological or psychiatric illness. Prior to eye-tracking, a subset of participants (aged ≥18) also completed a Montreal Cognitive Assessment (MoCA), a brief screening tool to detect mild cognitive impairment (Nasreddine et al., 2005). We excluded participants scoring below 20 on the MoCA, as lower cutoffs were recommended to reduce false-positive rates for mild cognitive impairment in diverse cohorts including older adults and lower education levels (Rossetti et al., 2011; Carson et al., 2017; White et al., 2022). MoCA cutoff and other exclusions are described in more detail in Yep et al. (2022), which used an overlapping set of participants. Written informed consent/assent was provided by all participants prior to the experiment. Recording sessions lasted ∼1 h, and participants were remunerated $20 CAD (or the equivalent via gift card) for their time.

### Eye-tracking apparatus

Data were collected using a video-based eye tracker (EyeLink 1000 Plus monocular-arm; SR Research) which recorded eye position monocularly at 500 Hz. Video-based eye-tracking is a noninvasive and sensitive method for capturing temporal changes in blink behavior (Sforza et al., 2008; Kwon et al., 2013; Nakano and Miyazaki, 2019). It permits analysis of large volumes of subjects, unlike manual counting techniques which are very time-consuming. Participants were seated in front of the eye tracker in a dark, windowless room, with the operator out of view. Their heads were supported with a chin and forehead rest, so that their eyes were at a fixed distance of 60 cm from the screen. All visual stimuli were displayed on a 17 inch LCD monitor (1,280 × 1,024 pixels, 32 bit color, 60 Hz refresh rate) controlled by the operator through a Dell Latitude E7440 Laptop. FV task movies were delivered at 30 fps using custom software in Ubuntu 13 to interface with the eye tracker via the SR Research API. Gaze position was calibrated and validated using a 9-point array (in 25 participants, eye-tracking was difficult, and a 5-point array was used instead). Calibration was intermittently verified using drift check and, if necessary, repeated at breakpoints in the experiments. An average validation error of <1.5° was considered sufficient for inclusion in analyses.

### Experimental design

#### Structured task: IPAST

**Figure 1.**
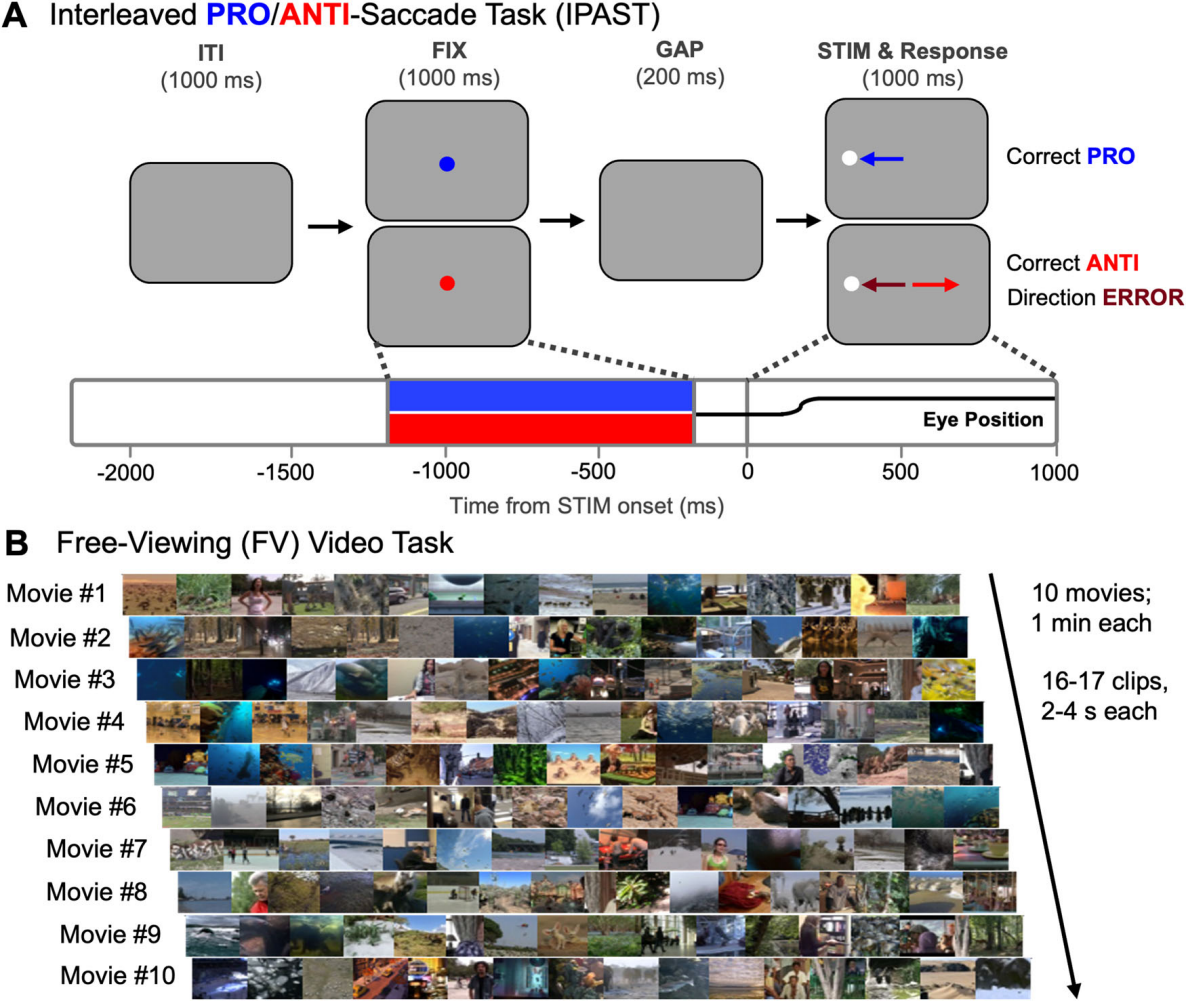
Schematics showing task design. ***A***, The interleaved pro-/anti-saccade task (IPAST). Each trial started with a central fixation cue (FIX), the color of which indicated the trial instruction (PRO saccade, blue; ANTI saccade, red). After a 200 ms GAP where the screen was black, a white peripheral stimulus (STIM) appeared at 10° horizontally to the left or right of where FIX had been. The participants were expected to generate a pro-saccade toward STIM or an anti-saccade away from STIM, based on the instruction they had been given by FIX. The example shows an eye position trace representative of a saccade being made during the appropriate epoch. Performing a pro-saccade on an anti-saccade trial was called a direction error. ***B***, The free-viewing (FV) Video Task. Participants watched 10 movies (each 1 min long) consisting of 16–17 individual clips (2–4 s duration each clip).

As described elsewhere (Yep et al., 2022), in the IPAST (Fig. 1*A*), each trial began with the appearance of a central fixation cue (0.5° diameter circle, 42 cd/m^2^) lasting 1,000 ms on a black background, the color of which indicated the saccade instruction (blue, pro-saccade; red, anti-saccade). This fixation epoch (FIX) was followed by a GAP period of 200 ms in which the screen was completely black, after which a peripheral target stimulus (STIM; 0.5° diameter circle; gray, 62 cd/m^2^) appeared for 1,000 ms at 10° horizontally to the left or right of screen center. Based on the instruction (as indicated by the FIX cue color), the participants were required to generate a pro-saccade toward the STIM, or instead suppress this automatic response and generate a voluntary anti-saccade away from the STIM, which requires inhibitory control. Each trial was separated by a 1,000 ms intertrial interval (ITI) consisting of a black screen, and the timing of all stimuli was verified using a photosensor. The saccade instructions (pro vs anti) as well as STIM locations (left vs right) were pseudorandomly interleaved. Participants completed two blocks of 120 trials, lasting a total of ∼20 min. We quantified behavior on trials with correct pro-saccades, correct anti-saccades, and direction errors, which are trials in which participants produced a pro-saccade in error on an anti-saccade trial. We did not quantify errors on pro-saccade trials, as they were extremely rare (∼1% of trials).

#### Unstructured task: FV

In the unstructured FV task, participants were instructed to simply “watch and enjoy” as they viewed 10 movies. The order of the first and last five movies was shuffled randomly between participants, but order of the clips within each movie remained fixed. The task was delivered in one continuous block with a total running time of 10 min. Each 1 min movie consisted of 16–17 clips (each 2–4 s in duration), resulting in 164 clips in total (Fig. 1*B*). Clips were not accompanied by audio and consisted of a variety of scenes containing people, nature, television, and cartoons. Clip content and length were randomized so that clip changes frequently introduced unpredictably timed perturbations of a large portion of the visual field providing new gaze targets (Tseng et al., 2013). Note that not all participants who participated in IPAST were able/willing to participate in FV.

### Data analysis

#### Preprocessing

All data were preprocessed using an automated analysis pipeline for blink and saccade detection (Coe et al., 2022) built in MATLAB (MathWorks). Blinks produce full or partial occlusion of the pupil, causing the video-based eye tracker to record a sudden loss of pupil data which is preceded and followed by large fluctuations in pupil area. Our task-agnostic algorithm (Coe et al., 2022) models the noise in this pupil area data to define the start and end of each blink.

To inform our definition of a viable blink, we inspected the durations of all pupil losses and used this to determine appropriate lower and upper bounds for blink durations. Data losses outside the chosen range were interpreted as tracking artifacts, or participants actively closing their eyes for longer periods, and were thus excluded from blink analysis. Note that throughout this paper, we have reported medians instead of means, to improve robustness to outliers without excluding participants based on their blink rate or average blink duration.

Outlier rejection was performed according to exclusion criteria outlined previously (Yep et al., 2022). Briefly, participants were excluded who had insufficient data to adequately characterize task performance (>40% of data contained eye loss), either due to poor eye-tracking or inability or unwillingness to complete the tasks. In the IPAST, we excluded participants with <30 viable trials (defined as those with a saccade taking place during the appropriate response window; between 90 and 800 ms) or >20% trials that were noncompliant (defined as those in which the participant never fixated, made a random saccade, or made no saccade at all). In FV, we excluded participants who viewed fewer than 10 movies.

#### Gaze clustering

Gaze clustering is a measure of how similar the viewing patterns were between participants. It was used to amalgamate several mechanisms related to engagement of attention, including saliency and individual interests or priorities, and evaluate their combined effect on blink behavior in the FV task. To obtain this measure, we first converted eye position of each subject to a Gaussian (∼2° diameter, 0.5° std) and then downsampled eye position in pixels to 1/10th (height, 103; width, 128). For each time sample (every 2 ms) starting from clip onset, we calculated the entropy (MATLAB function) of the group of eye positions and inverted the value to convert to gaze clustering. An average was computed for each clip and used to rank them on their degree of gaze clustering, from which a median split produced two groups: high and low gaze clustering (each containing 82 clips). We interpret high gaze clustering as more uniform engagement of attention among participants, and low gaze clustering as more randomness in scan paths between participants (i.e., less uniform engagement of attention). Blink probability was compared between these groups of clips using whole-trial and time-based analysis (see below, Statistical analysis).

#### Blink metrics

To calculate participant blink rate, we determined the number of viable blinks that occurred during the entire recorded period of the task and divided by the total recording time. This metric was useful for comparing between tasks using one value per participant.

Blink probability (the likelihood of the eye being closed at a given time point) was plotted as a continuous measure across time in a manner similar to previous studies (Siegle et al., 2008; Brych and Händel, 2020). Blink probability provides more temporal sensitivity than blink rate, as it acts as a two-dimensional index integrating both the frequency and length of the eye closure. Briefly, a logical array was produced spanning each data collection point (sampled at 500 Hz) which identified when the eye was open (0) or closed (1). In the IPAST, time was relative to stimulus onset and spanned 2,200 ms before and 1,000 ms after the event, yielding 240 arrays per participant (one per trial; each with 1,600 samples). These were combined to produce that individual's average blink probability across time aligned on stimulus onset. To assess the effect of differences in cognitive demand, separate averages were also produced for each type of trial (correct pro-saccade, correct anti-saccade, and direction error) for each participant.

In the FV task, time was relative to clip onset and spanned 500 ms before and 2,000 ms after the event, which corresponds to the length of the shortest clips. The first clip of each movie was not analyzed due to possible interference from preceding calibration breaks, resulting in 154 logical arrays per participant (one per analyzed clip; each with 1,100 samples). These were combined to produce that individual's average blink probability across time aligned on clip onset. Separate averages were produced for clips with high versus low gaze clustering for each participant. In both tasks, we also aimed to investigate sex differences in blink timing and whether they were driven by certain age groups. Therefore, we grouped participants by sex in the following age bins: 5–17, 18–44, and 45–93 years. These cutoffs were chosen to capture developmental and aging cohorts on the lower and higher ends, respectively.

### Statistical analysis

#### Blink rate as a function of sex and age

Generalized additive models (GAMs; Hastie and Tibshirani, 1986) were used to model blink rate as a continuous function of age in both tasks. As semiparametric regression models, GAMs are well-suited for the estimation of nonlinear trends across the lifespan while remaining robust to overfitting and variations in sampling of data points. These models are advantageous for studying age-related effects on behavioral measures because they do not assume known developmental and aging trajectories. GAMs have been previously used to model other eye-tracking measures across lifespan (Calancie et al., 2022; Yep et al., 2022).

We used the *mgcv* (Wood, 2017) and *LNCDR* (Tervo-Clemmens and Foran, 2022) packages in R to specify GAMs and perform change point analyses, respectively. To meet the assumption of normality for a Gaussian conditional distribution, we transformed blink rates using a Box-Cox transformation. Restricted marginal likelihood maximization was used to estimate smoothing parameters, **λ**, as it has been suggested to be the optimal approach (Wood, 2011). As described previously (Yep et al., 2022), statistically significant periods of change were then determined by estimating the first derivative and simultaneous 95% CI using posterior simulation. Statistically significant time points were defined as those where simultaneous confidence intervals of the first derivative did not contain zero (*p* < 0.05). Alongside age effects, we tested the inclusion of main effects of sex and determined statistically significant differences from time points where confidence intervals of the difference between smooths did not contain zero (*p* < 0.05). We then used the Bayesian information criterion (BIC) to compare goodness-of-fit between Model 1 (age) and Model 2 (age + sex), where lower values indicate better model performance.

#### Whole-trial analysis of blink probability

In the IPAST, we produced single-value averages for each subject's blink probability across (1) all trials, (2) correct pro-saccades, (3) correct anti-saccades, and (4) direction errors. A Spearman correlation was used to evaluate the association between average blink probability (all trials) and IPAST error rate (number of direction errors divided by total number of viable trials). A Kruskal–Wallis test (nonparametric one-way analysis of variance) was used to determine whether average blink probability varied based on trial type (correct pro-saccades vs correct anti-saccades vs direction errors). *P* values were adjusted via Bonferroni’s correction to control for multiple comparisons. In FV, we produced single-value averages for each subject's blink probability across (1) high gaze clustering clips and (2) low gaze clustering clips. A Wilcoxon signed rank test (nonparametric test for paired samples) was used to determine whether average blink probability varied between these gaze clustering groups. All statistical tests can be found in Table 1.

**Table 1.** Statistics table

Ref.	Figure	Data distribution	Type of test	Power
a		Non-normal	Kruskal–Wallis	*η*^2 ^= 0.01
b		Non-normal	Dunn post hoc test	Diff: 105.808 ± 70.564
c		Non-normal	Dunn post hoc test	Diff: −111.608 ± 70.564
d		Non-normal	Spearman correlation	*ρ *= 0.100
e		Non-normal	Wilcoxon signed rank	*z*-val = −10.743
f	8*A*, left	Non-normal	Wilcoxon signed rank	*z*-val = 7.115
g	8*A*, right	Non-normal	Spearman correlation	*ρ *= 0.687
h	8*B*, left	Non-normal	Wilcoxon signed rank	*z*-val = 11.468
i	8*B*, right	Non-normal	Spearman correlation	*ρ *= 0.598
j	8*C*, right	Non-normal	Spearman correlation	*ρ *= 0.496

#### Time-series analysis of blink probability

Probabilistic blink time-series present unique challenges for statistical analysis. The relative scarcity of blinks results in many values being near zero, often violating the assumptions of normality imposed by parametric tests and producing too many tied values to permit certain nonparametric approaches (e.g., Mann–Whitney *U*). Furthermore, the dense sampling in our time-series (every 2 ms) produces substantial autocorrelation; measurements at adjacent time windows are very closely related as the function reflects gradual changes over many trials averaged together. This is an important caveat for determining family-wise error rate when accounting for multiple comparisons. To address these challenges, we developed a workflow loosely resembling that proposed by Seedorff et al. (2018), which is adapted to detect precise time intervals where blink probability curves differ.

First, cubic smoothing splines were fit to the raw participant data (csaps function in MATLAB, smoothing factor of 0.00001), as it was suggested to use any nonlinear fit to minimize the appearance of spurious regions of significance or insignificance caused by noise in individual curves (Seedorff et al., 2018). Next, we used a bootstrapping approach to estimate the mean and 95% CIs (type, student) by resampling randomly with replacement from the fitted participant curves (Efron, 1982). After 1,000 bootstrap iterations, the resampled means were averaged to produce the population- and group-level curves for plotting. To obtain group-level comparisons (e.g., female vs male; correct pro- vs correct anti-saccade), the difference of means and their 95% CIs were then calculated at each time point. This method substitutes traditional hypothesis testing because, if the 95% CI for the difference score does not include 0, we can consider those groups to be significantly different from one another at that time point (Gardner and Altman, 1986). Furthermore, to account for multiple comparisons and reduce the risk of false periods of significance, we leveraged a form of permutation testing by sampling an unlabeled version of our data without replacement (e.g., testing for significant differences after randomly splitting participants into two equal groups to simulate a spurious sex difference). For each permutation, we stored the duration of the longest period of significant difference (consecutive millisecond of a false-positive result), which can be interpreted as differences driven by noise and autocorrelation in the time-series data. After 1000 permutations, we calculated the upper 95% CI of this estimate to obtain our cutoff (Table 2). We filtered our measures of significance for each comparison accordingly, by only reporting differences at times where blink probability was significantly different for *at least* that duration of consecutive millisecond in a row.

**Table 2.** Cutoffs for statistical significance obtained from permutation analysis

Task	Comparison	Cutoff for significance (ms)
IPAST	Pro-saccade to anti-saccade	200
	Anti-saccade to direction error	200
	Pro-saccade to direction error	200
	Sex (all ages)	228
	Sex (<18)	290
	Sex (18–45)	344
	Sex (>45)	316
FV	High to low gaze clustering	138
	Sex (all ages)	218
	Sex (<18)	266
	Sex (18–45)	212
	Sex (>45)	201

Cutoffs are rounded up to the nearest even number. They indicate the minimum consecutive duration for which two curves must be significantly different to assume a true effect in each comparison. IPAST, interleaved pro-/anti-saccade task; FV, free-viewing task.

#### Comparison between tasks

To parse differences driven by task structure, we compared overall blink rates, median blink durations, and interblink intervals between tasks for participants who completed both the IPAST and FV. Interblink intervals are the time elapsed between the end of one blink and the onset of the next. Given the non-normal distribution of blink parameters, Wilcoxon signed rank tests were used to evaluate differences between task means, and Spearman correlations were run to investigate pairwise associations for each parameter (Table 1).

### Code accessibility

Available upon request.

## Results

### Data loss durations

**Figure 2.**
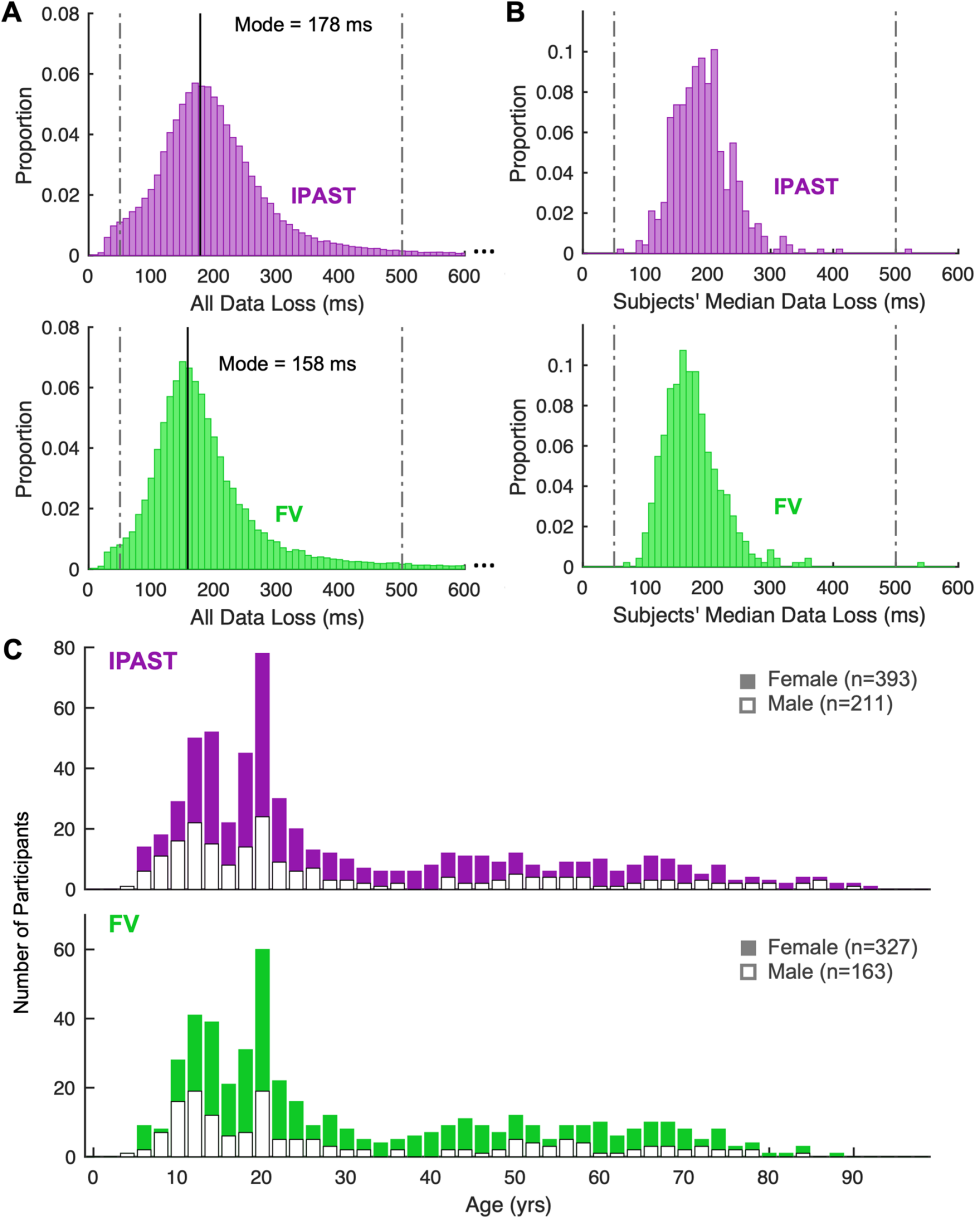
Raw blinks and study cohort. ***A***, Duration of all data loss periods. Bin width is 10 ms. Dotted lines at 50 and 500 ms correspond to the cutoffs determining whether the data loss is interpreted as a blink. ***B***, Median data loss duration of each subject. Bin width is 10 ms. Dotted lines at 50 and 500 ms correspond to the cutoffs determining whether the data loss is interpreted as a blink. ***C***, Age and sex distribution of participants in each task. Bin width is 2 years.

We observed that 95% of data losses in the IPAST and 92% in FV had durations between 50 and 500 ms, which are supported by previous literature quantifying blink duration (Fig. 2*A*; Caffier et al., 2003; Divjak and Bischof, 2009). Furthermore, after plotting the distribution of each subject's median data loss duration (Fig. 2*B*), we confirmed that a cutoff of 500 ms was unlikely to skew the data, given only one subject had a median of >500 ms.

### Study cohort

From 2015 to 2022, 631 individuals (409 F, 5–93 years of age) participated in the IPAST, and 523 individuals (345 F, 5–91 years of age) participated in FV. After exclusions (Table 3), 604 participants (393 F) remained for IPAST and 490 participants (327 F) for FV, of which 475 (319 F) completed both tasks. Additional details on sex and age distribution of included participants can be found in Table 4 and Figure 2*C*. We note that the cohort is skewed toward young females (<25 years old) due to extensive participant recruitment from local university and college student bodies in efforts to age-match control participants to various neuropsychiatric patient cohorts within this demographic.

**Table 3.** Reasons for excluding each participant

Task	Reason	Num. of participants excluded
IPAST	MoCA score <20	3
	PRO or ANTI viable trial count <30	10
	PRO or ANTI noncompliance trial count >20%	14
FV	MoCA score <20	2
	<10 movies completed	15
	>40% of data contained eye loss	4
	Average validation error of >1.5°	11
	No demographic information provided	1

IPAST, interleaved pro-/anti-saccade task; FV, free-viewing task; PRO, pro-saccade trial; ANTI, anti-saccade trial.

**Table 4.** Summary of demographic information for included participants

Task	Age bin (*n*)	Mean age ± SD	Sex (*n*)	Education level (*n*)
IPAST	5–93 (604)	F: 32.4 ± 20.9 M: 30.6 ± 22.5	F (393) M (211)	Elementary (112) High School (127) College/Trade (72) Undergraduate (184) Graduate (87) Professional (17)
	5–17 (185)	F: 13.2 ± 2.8 M: 12.2 ± 2.9	F (106) M (79)	Elementary (112) High School (72) College/Trade (1)
	18–44 (261)	F: 26.5 ± 8.0 M: 25.1 ± 6.9	F (184) M (77)	High School (36) College/Trade (24) Undergraduate (135) Graduate (57) Professional (6)
	45–93 (158)	F: 63.1 ± 12.0 M: 64.8 ± 12.5	F (103) M (55)	High School (19) College/Trade (48) Undergraduate (50) Graduate (30) Professional (11)
FV	5–93 (490)	F: 33.5 ± 20.7 M: 30.9 ± 21.7	F (327) M (163)	Elementary (86) High School (93) College/Trade (68) Undergraduate (147) Graduate (77) Professional (15)
	5–17 (147)	F: 13.4 ± 2.8 M: 12.5 ± 2.7	F (84) M (63)	Elementary (86) High School (60) College/Trade (1)
	18–44 (202)	F: 26.7 ± 8.2 M: 25.5 ± 6.8	F (148) M (54)	High School (18) College/Trade (20) Undergraduate (109) Graduate (47) Professional (4)
	45–93 (141)	F: 62.0 ± 10.7 M: 62.4 ± 10.0	F (95) M (46)	High School (15) College/Trade (47) Undergraduate (38) Graduate (30) Professional (11)

IPAST, interleaved pro-/anti-saccade task; FV, free-viewing task; F, female; M, male.

### Differences across age as a continuous measure

**Figure 3.**
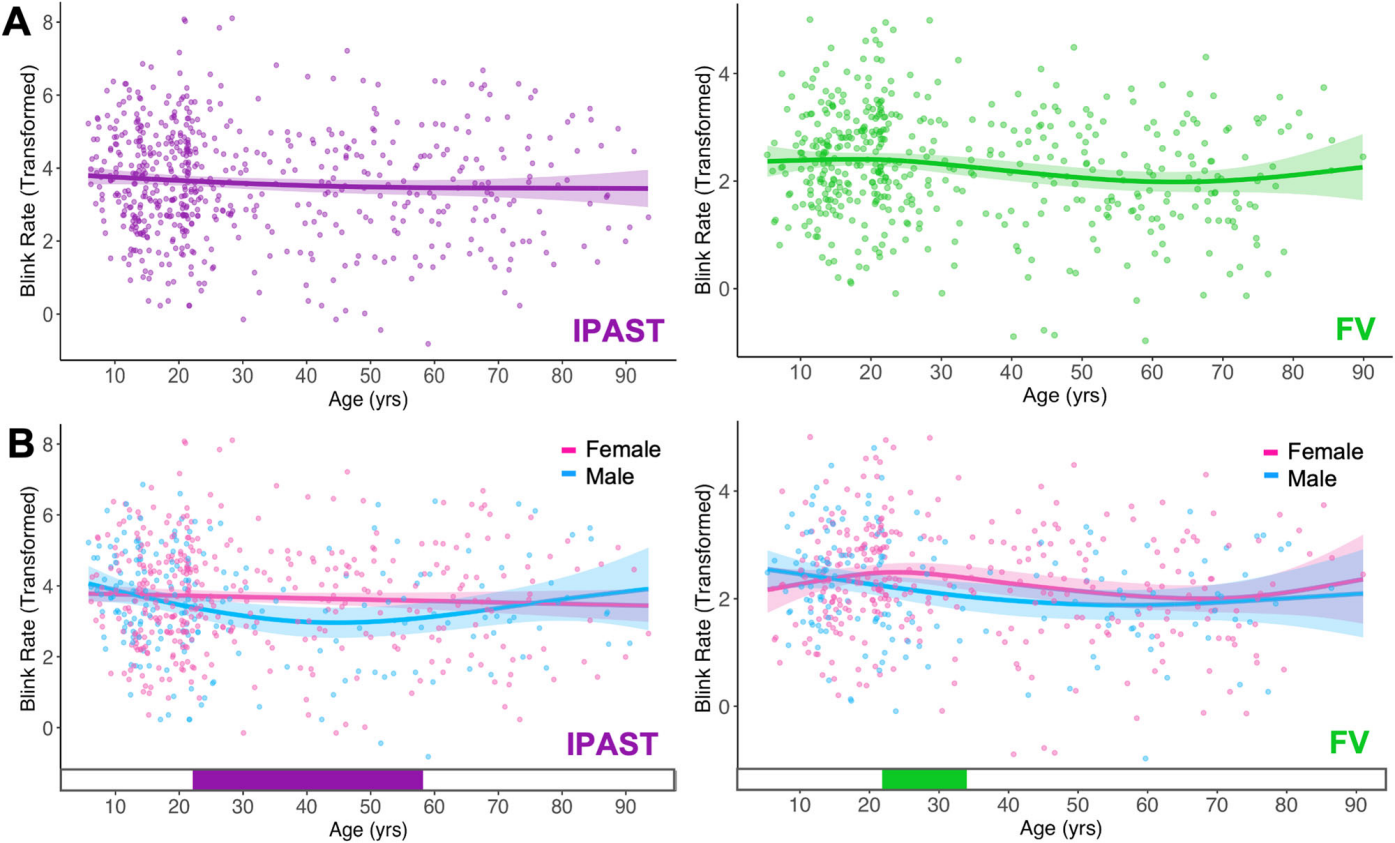
GAM fits and significant periods of change in IPAST (left) and FV (right). Scatter points are individual subjects, curves are the GAM fits colored accordingly, and shaded ribbons are the 95% CIs. ***A***, Blink rate modeled as a continuous function of age. There were no significant periods of age-related change in either task. ***B***, Blink rate modeled as a continuous function of age, with participant sex as a factor. The bottom tile indicates the ages where sexes significantly differ (22–58 years in IPAST; 22–34 years in FV).

GAMs for smoothed fixed effect of age were specified to investigate whether blink rate changed across lifespan (Model 1; Fig. 3*A*). Change-point analysis revealed no periods of significant age-related change in either task. Next, GAMs defined by smoothed effect of age split by sex were specified to investigate the effects of participant sex on blink rates (Model 2; Fig. 3*B*). Males had lower blink rates than females between the ages of 22 and 58 in the IPAST (*R*^2^ of 0.017) and between the ages of 22 and 34 in FV (*R*^2^ of 0.0357). All GAM fit parameters are displayed in Table 5 (*ref df*, *F*, *p*, *R*^2^, deviance explained, BIC). Model 1 had a slightly better fit than Model 2 in both tasks, although this is likely due to BIC favoring models that are less complex (Wagenmakers and Farrell, 2004).

**Table 5.** GAM fit parameters

Task	Model	Ref df	*F*-val	*p*-val	*R* ^2^	Deviance explained	BIC
IPAST	1	1.867	1.443	0.174	0.004	0.665%	2,234.115
	2 (F, M)	1.002 3.138	1.136 3.140	0.287 0.0236	0.017	2.43%	2,243.539
FV	1	3.492	4.204	0.0043	0.028	3.31%	1,427.033
	2 (F, M)	4.099 2.397	2.668 3.237	0.0298 0.0284	0.0357	4.78%	1,444.309

Model 1 is a continuous function of age, and Model 2 adds sex as a factor. BIC indicates goodness of fit; the lower the value, the better the fit. IPAST, interleaved pro-/anti-saccade task; FV, free-viewing task; F, female, M, male.

### Blink probability throughout IPAST

**Figure 4.**
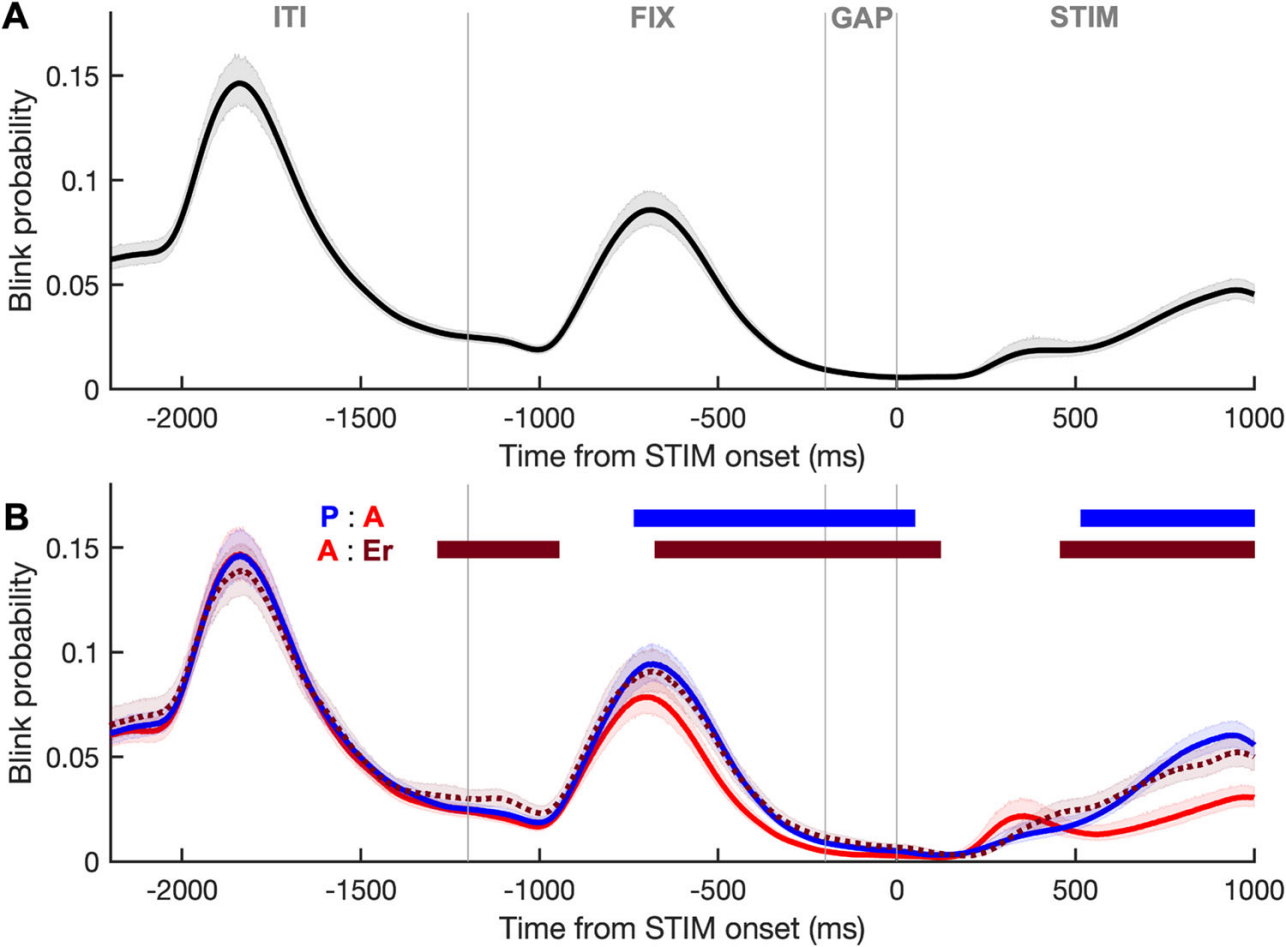
Blink behavior in IPAST. ***A***, Blink probability relative to STIM onset (at 0 ms) across all IPAST trials. Trial length includes the ITI (−2,200 to −1,200 ms), fixation epoch (FIX; −1,200 to −200 ms), GAP (−200 to 0 ms), and stimulus epoch (STIM; 0 to 1,000 ms). Shaded area represents the 95% CIs. ***B***, Blink probability relative to STIM onset, separated by trial type (PRO, blue line; ANTI, red line; DIRECTION ERROR, dotted maroon line). Horizontal bars represent periods where curves differ significantly, labeled by the comparison made (P:A, PRO vs ANTI; A:Er, ANTI vs DIRECTION ERROR) and colored according to the trial type with the higher blink probability at that time. Shaded area represents the 95% CIs.

To investigate blink timing relative to the appearance of predictable stimuli, we calculated blink probability across all trials in the IPAST (Fig. 4*A*). Blinks were most likely to occur during “low risk” periods where participants had already obtained relevant information and knew nothing new would be happening. For example, blink probability spiked during the middle of the ITI when the screen was blank and, to a lesser degree, in the middle of the FIX epoch after they had just received the trial instruction. Blinks were least likely in the time leading up to and immediately following STIM onset and then increased following the saccade response.

Next, we compared correct pro-saccade, correct anti-saccade, and direction error trials to determine if blink timing varied by IPAST trial type, which would implicate inhibitory control mechanisms. As expected, whole-trial blink probability varied by trial type (*χ*^2^_(2)_ = 17.42; *p *= 1.650 × 10^−4a^; *η*^2 ^= 0.01; Kruskal–Wallis test; with a median of 0.037 for correct pro-saccades, 0.032 for correct anti-saccades, and 0.038 for direction errors). Both correct pro-saccade (*p *= 0.0013^b^; post hoc) and direction error (*p *= 0.0006^c^; post hoc) trials produced significantly higher blink probabilities than correct anti-saccade trials. Detailed temporal analysis revealed that the time periods driving this difference occurred from the middle of FIX to STIM onset and again after the saccade was made (stimulus-driven saccades start after 90 ms), where both correct anti-saccade trials had significantly higher blink probability than both other types (Fig. 4*B*; Cohen's *d*, ∼0.2–0.4). Direction error trials did not differ from correct pro-saccade trials at any point but had higher blink probability than correct anti-saccade trials during the appearance of the FIX cue. IPAST error rate was only very weakly correlated to participants’ overall blink probability (*ρ *= 0.100; *p *= 0.014^d^; Spearman), suggesting that time-based analyses are more informative.

#### Sex differences in IPAST blink timing

**Figure 5.**
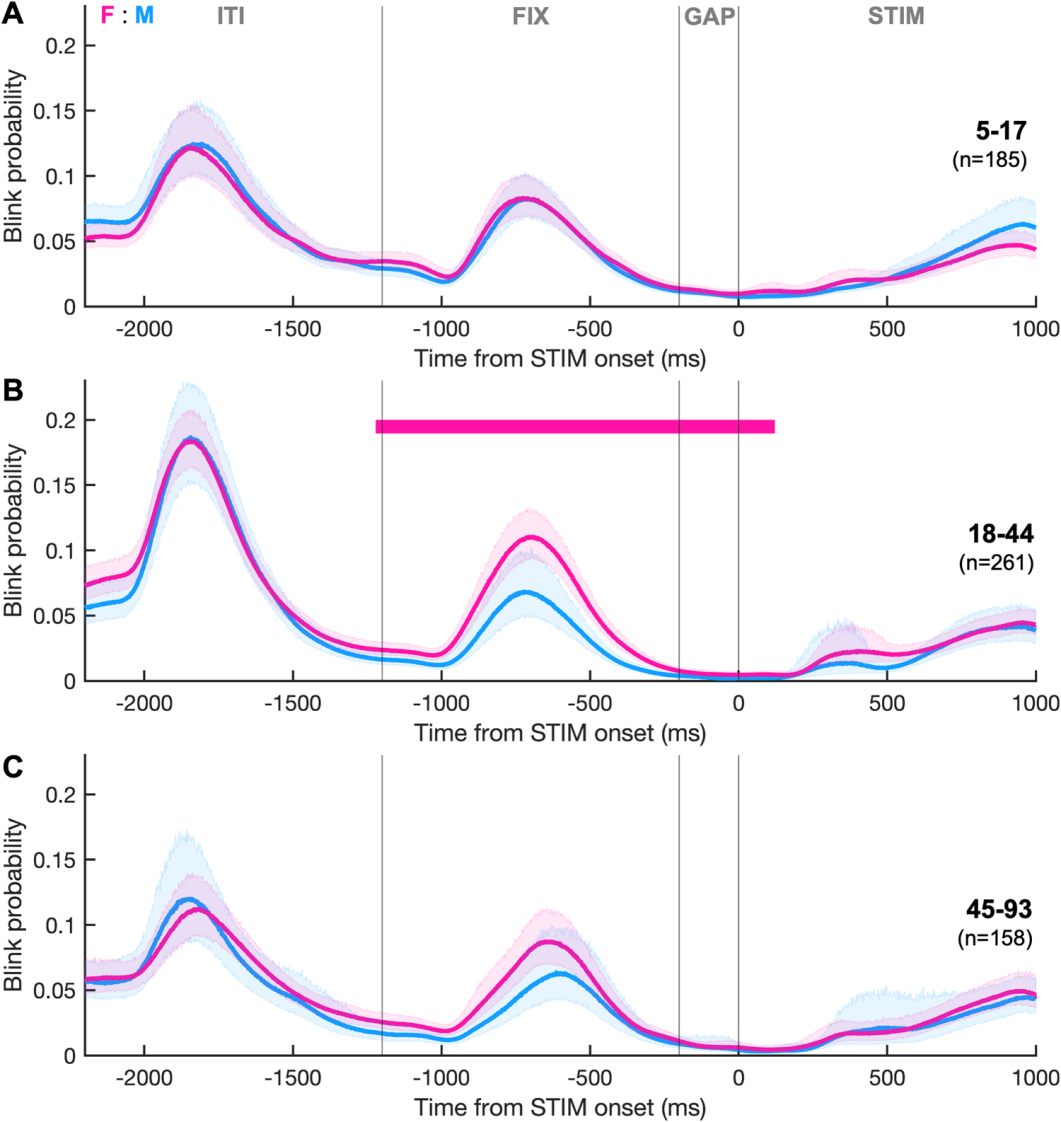
Sex and age differences in IPAST. Blink probability relative to STIM onset compared between sexes within different age bins: (***A***) 5–17, (***B***) 18–44, and (***C***) 45–93 years of age. The horizontal bar indicates periods where curves differed significantly between sexes and is colored pink given that females had a higher blink probability. Shaded area represents the 95% CIs.

To determine whether blink timing varied by sex in IPAST, we compared blink probability between sexes within discrete age bins. In the middle age group (18–44 years), females had a significantly higher probability of blinking for the entirety of the FIX epoch (Fig. 5*B*; Cohen's *d*, ∼0.3–0.5). No significant differences were found between sexes for age bins of 5–17 and 45–93 years (Fig. 5*A*,*C*).

### Blink probability throughout FV

**Figure 6.**
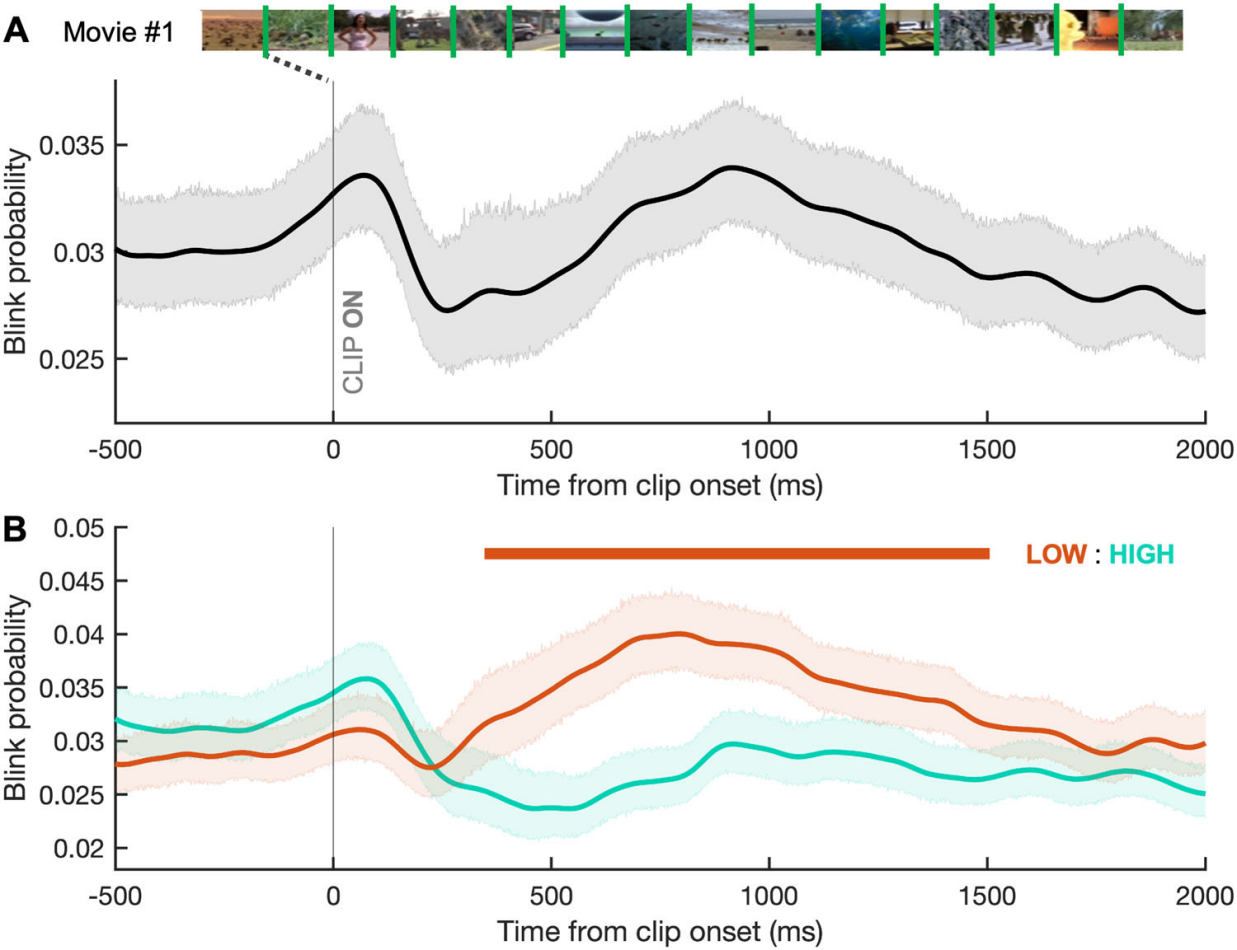
Blink behavior in FV. ***A***, Analysis of blink probability relative to clip onset across all FV movies. Movie #1 shown across the top, with clip changes indicated with green lines between the 16 clips. Shaded area represents the 95% CIs. ***B***, Blink probability relative to clip onset in FV, separated by the degree of gaze clustering of the new clip (HIGH, teal line; LOW, orange line). Horizontal bars represent periods where curves differ significantly and are colored according to the clip type with the higher blink probability at that time. Shaded area represents the 95% CIs.

To investigate how blinking changed in response to scene changes in FV, we calculated blink probability relative to clip onset for all participants across all movies (Fig. 6*A*). This revealed three important behaviors: (1) a strong inhibition of blinking after the clip change, then (2) a gradual rebound of blinking peaking halfway through the clip, followed by (3) a gradual decline in blinking for the remainder of the clip. Clips were also split into high and low gaze clustering groups, scored by how close together participants’ scan paths were across the length of the clip. Whole-trial blink probability differed by degree of gaze clustering, with high gaze clustering clips having a significantly lower blink probability than low gaze clustering clips (*p *= 9.98 × 10^−26e^; Wilcoxon signed rank). Figure 6*B* localizes this difference as a significantly stronger blink suppression on high gaze clustering clips lasting from 352 to 1,510 ms (Cohen's *d*, ∼0.2–0.4).

#### Sex differences in FV blink timing

**Figure 7.**
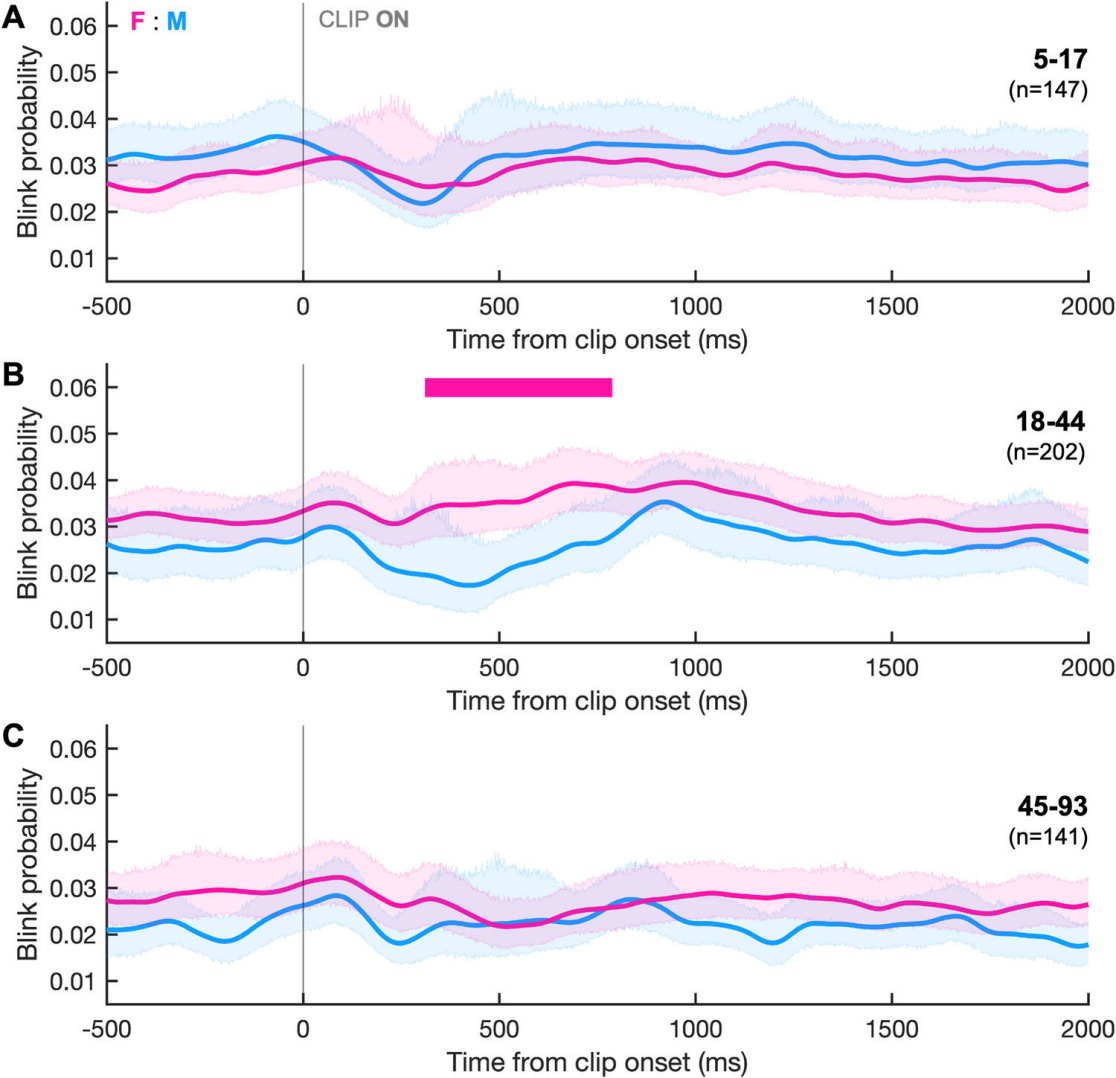
Sex and age differences in FV. Blink probability relative to STIM onset compared between sexes within different age bins: (***A***) 5–17, (***B***) 18–44, and (***C***) 45–93 years of age. The horizontal bar indicates periods where curves differed significantly between sexes and is colored pink given that females had a higher blink probability. Shaded area represents the 95% CIs.

**Figure 8.**
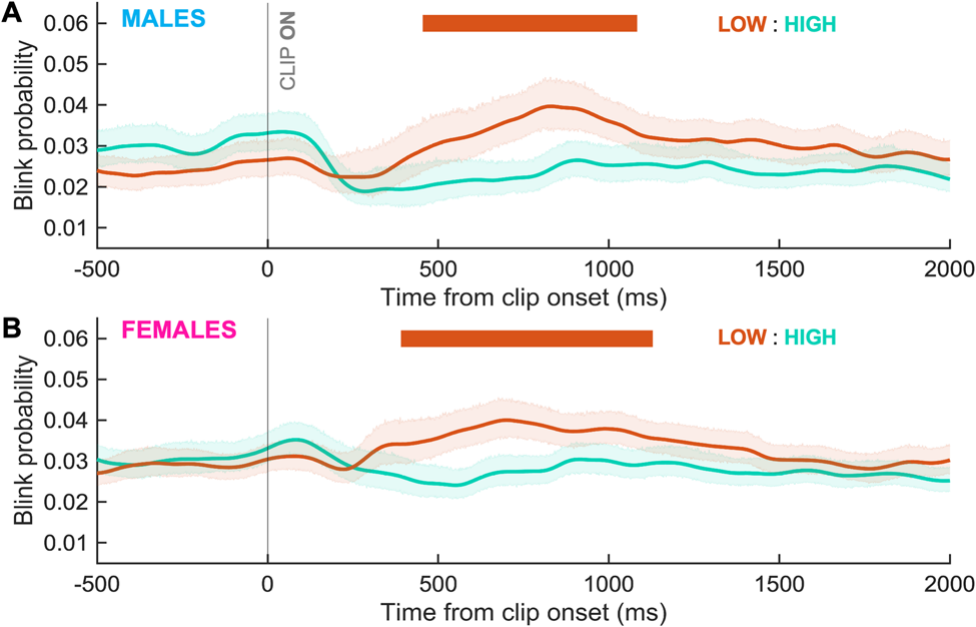
Gaze clustering effect by sex. Blink probability relative to STIM onset compared between high and low gaze clustering clips separately for (***A***) males and (***B***) females. The horizontal bar indicates periods where curves differed significantly between clip types and is colored orange given that low gaze clustering clips had a higher blink probability. Shaded area represents the 95% CIs.

To determine whether blink timing varied by sex in FV, we compared blink probability between sexes within discrete age bins. In the middle age group (18–44 years), females had a significantly higher probability of blinking for a brief period after clip change (Fig. 7*B*; Cohen's *d*, ∼0.4). There were no significant differences between sexes for the age bins of 5–17 and 45–93 years (Fig. 7*A*,*C*). The gaze clustering effect occurred in both males (Fig. 8*A*; Cohen's *d*, ∼0.2–0.6) and females (Fig. 8*B*; Cohen's *d*, ∼0.2–0.4).

### Comparison of blink metrics between tasks

**Figure 9.**
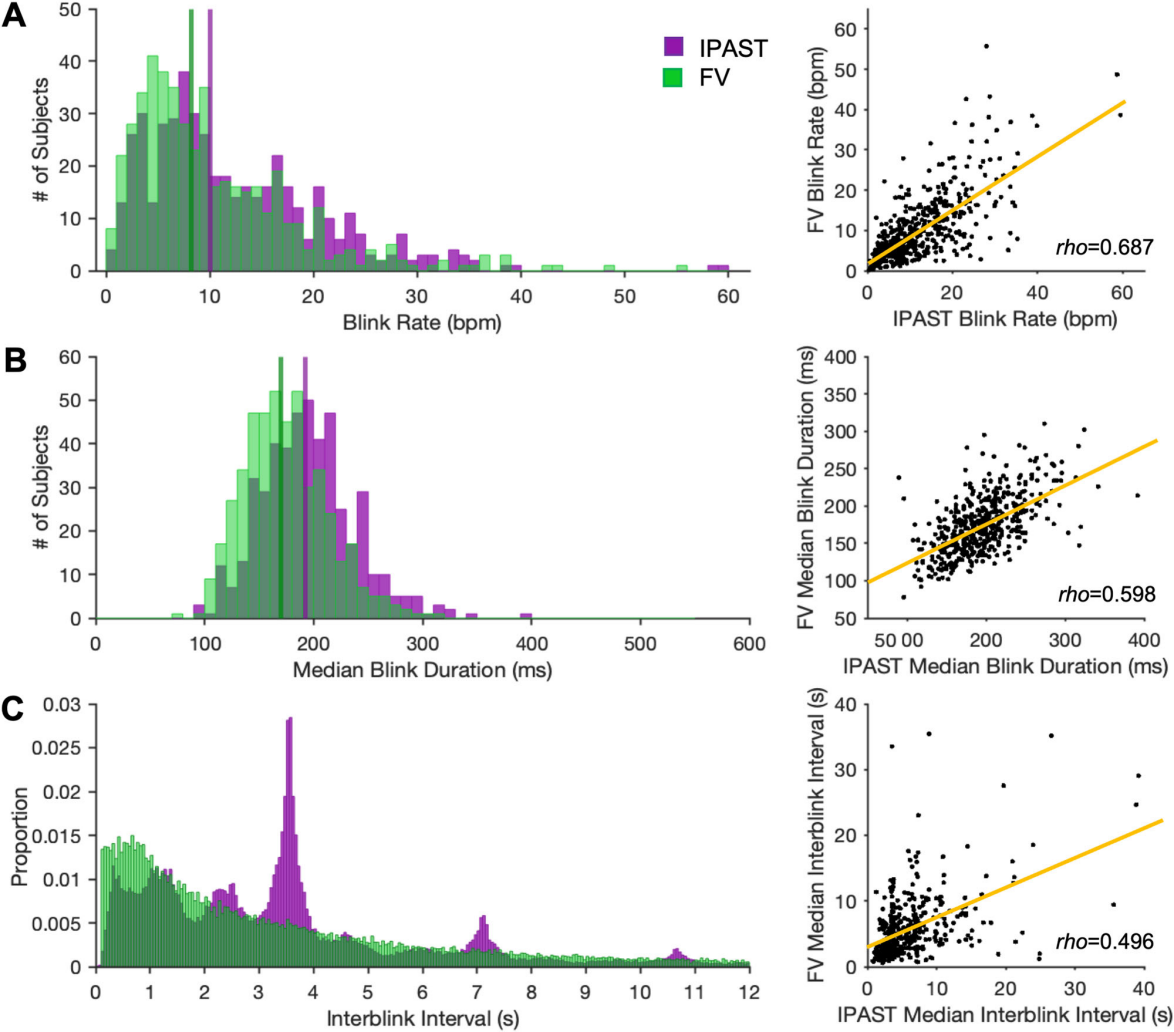
Comparison of blink metrics between tasks. Blink rates (***A***) and median blink durations (***B***) were compared using histograms (left panels) and Spearman correlations (right panels). IPAST demonstrated significantly higher blink rates (*p *= 1.162 × 10^−05^) and durations (*p *= 1.903 × 10^−30^) than FV. Blink rates showed a moderate positive correlation between tasks (*ρ *= 0.687, *p *= 0.00), as did blink durations (*ρ *= 0.598, *p *= 2.043 × 10^−47^). Yellow lines model the line of best fit to the data (***A***, *y* = 0.67x + 2.1; ***B***, *y* = 0.50x + 80.6). ***C***, Comparison of all interblink intervals, not averaged by subject (left), and Spearman correlation of subjects’ median interblink intervals between tasks (right). Median interblink intervals showed a moderate positive correlation between tasks (*ρ *= 0.496; *p *= 1.132 × 10^−30^). Yellow line models the line of best fit to the data (*y* = 0.49x + 4.8).

We next compared blink metrics between the IPAST and FV tasks. Blink rates and median blink durations were highly variable between participants, ranging from 0.2 to 59 blinks per minute (bpm) and 78 to 392 ms, respectively. Median blink rate across all participants was 10.0 (±7.2) bpm in the IPAST and 8.2 (±6.0) bpm in FV (Fig. 9*A*). Median blink duration after preprocessing cutoffs (see Materials and Methods) was 192 (±34) ms in the IPAST and 170 (±30) ms in FV (Fig. 9*B*). Participants had significantly higher blink rates (*p *= 1.162 × 10^−5f^) and longer median blink durations (*p *= 1.903 × 10^−30g^) in the structured IPAST. Panels on the right in Figure 9 display moderate positive associations for blink rates (*ρ *= 0.687; *p *= 0.00^h^), median blink durations (*ρ *= 0.598; *p *= 2.043 × 10^−47i^), and median interblink intervals (*ρ *= 0.496; *p *= 1.132 × 10^−30j^) between tasks.

Figure 9*C* (left) shows the distribution of interblink intervals occurring in each task. In the IPAST, interblink interval distribution was multimodal due to blinking at discrete times in the structured task. The largest peaks occurred at ∼3.5 s (blink every trial), ∼1 s, and ∼2.5 s. There were smaller peaks at ∼7 s (blink every two trials) and ∼10.5 s (every three trials). In FV, the distribution was more uniform, demonstrating mostly short interblink intervals of 0–2 s, due to blink timing being less restricted by task structure.

## Discussion

Spontaneous blinking has gained momentum as an indirect measure in various cognitive domains, yet substantial interindividual variability complicates the story. We aimed to fill this gap in knowledge by leveraging a large cohort of 604 healthy participants using more temporally sensitive analytical methods. Given the extreme task sensitivity of blink behavior (Stern et al., 1984; Goldstein et al., 1985), we sought to clarify the relationship to internal mental processes using two distinct paradigms that were contrasted for cognitive difficulty and perceptual load. Crucially, we demonstrated that blinking had a more complex relationship to sex and age than was previously described. Blink behavior was also remarkably sensitive to task demands and adaptive to fluctuations in visual processing, attentional engagement, and inhibitory control requirements. Together, these findings provide much-needed normative data across the lifespan and highlight several important considerations for blink research; tasks should be carefully designed to reduce confounds arising from distinct yet related mental processes, and sex and age should be analyzed in more robust and time-based ways to capture their effect.

### Mechanisms underlying blink behavior

#### Visual processing

Blink behavior was remarkably consistent across all participants in the IPAST. Blink probability decreased in preparation for stimuli appearing on-screen at predictable times (Fig. 4*A*). Blinks increased briefly after receiving the trial instruction during FIX and again after STIM onset but did not peak until the ITI separating the trials. These findings align with previous work showing that blink rate decreases during stimulus processing and briefly rebounds immediately afterward, reaching a maximum after trial completion when information is released from working memory (Fukuda, 2001; Ichikawa and Ohira, 2004). Given the mental engagement necessary between FIX onset and STIM disappearance, this also reinforces the idea that blinking flanks periods of increased cognitive load (Siegle et al., 2008; Wascher et al., 2015). Auditory signals have been found to modulate blink behavior in a similar fashion, supporting that temporal blink patterns are not dependent on stimuli being visual and instead represent internal mental processes (Bauer et al., 1985; Kobald et al., 2018; Huber et al., 2022).

The FV task revealed blinking strategies for processing of continuous visual input. Blink probability dropped sharply after each clip change as viewers oriented to novel stimuli and then rebounded gradually before decreasing again toward the end of the clip (Fig. 6*A*). The start of this rebound period coincides with the previously reported latencies of the first blink after a scene break while watching movies (Nakano et al., 2009). It is likely that blink suppression after clip change facilitates evaluation of new visual targets, after which completion of visual processing induces a rebound.

#### Attentional engagement

After a brief initial period of blink suppression for both types of FV clips, high gaze clustering clips maintained a low blink probability while low gaze clustering clips produced a prominent rebound (Fig. 6*B*). We interpret gaze clustering as whether attention is engaged less or more uniformly across the participants, making this consistent with previous findings of blinks being synchronized between individuals at attentional lulls in movies (Nakano et al., 2009). In fact, blink rates have been suggested to index attentional engagement (Maffei and Angrilli, 2018, 2019), individual interest (Ranti et al., 2020), and perceived stimulus importance (Shultz et al., 2011), which were all likely contributing to the gaze clustering measure. We propose that in fast-paced movie formats, clips are quickly evaluated as being engaging or not (automatic visual processing), after which blink probability decreases or increases to match the demand on attention. This adjustment is made subconsciously, as participants had no ability to predict clip onset given the randomized clip lengths and no prior expectations of upcoming clip content.

#### Inhibitory control

Executing a correct anti-saccade requires suppression of the automatic response of looking toward a visually salient stimulus, followed by the voluntary execution of a saccade in the opposite direction (Hallett, 1978). Failure to suppress the automatic response, resulting in a pro-saccade on an anti-saccade trial, is termed a direction error (Munoz and Everling, 2004). IPAST error rate is an established behavioral measure of inhibitory control and executive function (Yep et al., 2018, 2022) and was weakly correlated to blink probability in this study. Correct anti-saccade trials had significantly lower blink probability in the FIX and STIM epochs than both pro-saccade and direction error trials (Fig. 4*B*). This indicates that the heightened inhibitory signals necessary for suppression of the pro-saccadic response on an anti-saccade trial may share the same neural mechanisms that suppress blinks until the appropriate saccade has been made. Trial success may therefore be tied to achieving a threshold level of blink suppression well in advance of STIM appearance, indicative of adequate inhibitory control on that trial. This supports the idea that higher blink rates are predictive of less efficient inhibitory processing (Colzato et al., 2009).

### Sex and age-related mechanisms underlying blink behavior

This is the first time blink rates have been thoroughly analyzed as a continuous function of age across the entire lifespan. GAMs revealed there were no specific periods of age-related change in either task (Fig. 3*A*). Blink rates differed significantly by sex between the ages of 22 and 58 in IPAST and 22 and 34 in FV (Fig. 3*B*). The discrepancy between the IPAST and FV tasks indicates that experimental context has some interaction with demographic factors (Bentivoglio et al., 1997). Given the multitude of contradictions in the literature on sex differences in blink rate (Dalcegio et al., 2012; Coors et al., 2021; Mbamba et al., 2023), we propose that modeling sex effects continuously across lifespan is a more reliable approach than analyzing discrete age bins.

In the IPAST, females (aged 18–44) had higher blink probabilities during the presentation of the FIX instruction than males of the same ages (Fig. 5*B*), yet their performance accuracy and reaction times did not differ (Yep et al., 2022). This is consistent with a previous study that showed females had higher blink rates than males during a fixation task (Coors et al., 2021). Females (aged 18–44) also had higher blink probability immediately after the clip change in FV (Fig. 7*B*). One possible explanation is that blink suppression relates to maintaining vigilance, a responsibility falling primarily on adult males in primate social groups, and therefore may have become sex-linked through evolution (Matsumoto-Oda et al., 2018). However, sex has a complex relationship with gender, making it difficult to classify these differences as inherently biological rather than social, especially given the vast gender differences in visual processing strategies (Darley and Smith, 1995; Gomez et al., 2019) and nonverbal communication through eye contact (Spangler, 1995).

### Comparison of blink rates and durations between tasks

Blink rates and durations were significantly lower during the FV task despite it taking place later in the experiment, following the more difficult IPAST. This is strong evidence that higher perceptual load drives blink suppression independent of task difficulty (Cardona et al., 2011), especially considering that fatigue and prolonged use of a computer screen would generally have been expected to increase blink rate and duration (Patel et al., 1991; Stern et al., 1994). FV requires the participant to attend to a continuous stream of complex visual stimuli, while in the IPAST, attention is engaged in discrete blocks due to ITIs being present between trials. Interblink intervals were more rigid in IPAST than those in FV, reflecting a tendency to blink during fixed, periodically occurring windows. As supported by the distribution of interblink intervals in Figure 9*C* (left), individuals with lower blink rates likely blink during the ITI once per trial or every couple of trials, while those with higher blink rates leverage multiple windows of decreased attentional demand, such as both the ITI and FIX.

### Limitations

This study represents the most comprehensive analysis of sex, age, and experimental context on blink behavior to date, which is important for understanding the factors driving healthy interindividual variability so that truly abnormal oculomotor behavior can be accurately characterized as such. Although potential confounds, we saw value in preserving the natural variability in our data as it incorporates the multitude of factors which cannot always be controlled for in real-world conditions or ignored as outliers: inherent biology, social determinants, mental state, and other physical (makeup, contact lenses) or environmental factors (humidity, smoky weather conditions).

### Future directions

So far, our lab has used the tasks described in this paper to differentiate saccade and pupil dynamics in multiple neurodegenerative and psychiatric cohorts (Wang et al., 2016; Perkins et al., 2021; Habibi et al., 2022; Riek et al., 2023), making blinks an obvious next candidate for use in detecting clinically relevant deficits in cognitive pathways, especially given the findings described here. Alterations in blink behavior have already been observed in many neurological conditions (Karson et al., 1984; Basso et al., 1996) but would benefit from the more temporally sensitive, probabilistic approaches to blink analysis implemented in this study to expose the higher-level mechanisms underlying the behavior (Brien et al., 2023; Calancie et al., 2023).

## Synthesis

Reviewing Editor: Nicholas J. Priebe, University of Texas at Austin Decisions are customarily a result of the Reviewing Editor and the peer reviewers coming together and discussing their recommendations until a consensus is reached. When revisions are invited, a fact-based synthesis statement explaining their decision and outlining what is needed to prepare a revision will be listed below. The following reviewer(s) agreed to reveal their identity: NONE. Note: If this manuscript was transferred from JNeurosci and a decision was made to accept the manuscript without peer review, a brief statement to this effect will instead be what is listed below.

Two reviewers evaluated the manuscript "Attentional modulation of eye blinking is altered by sex, age, and task structure" and both reviewers agree that the authors have provided an important description of the nature of eye blinking during both free viewing and common oculomotor tasks. The authors were able to take advantage of their database to uncover the degree of blinking behavior across subjects, across age and across sex. The reviewers raised a few issues to improve the presentation of this study. These suggestions are detailed below, which you should find useful in revising your manuscript, if you decide to resubmit it to eNeuro or to another journal.

Issues:

1) In the IPAST task did timing of blink prior to stimulus onset have an impact on SRT of correct pro- and anti-saccades?

2) Since pupil diameter data was likely collected during these experiments, is there a consistent modulation in pupil diameter relative to blink timing.

3) It will be interesting to see the figure 7 results separately for high and low gaze clustering clips. In the current figure it seems all the post clip onset suppression effect is due to male participants.

4) Development. Since there are many data points for the ages ranging between 5-18 years, is it be possible to divide this group in smaller age bins to look at any developmental patterns?

5) Manuscript organization. It is unclear why figure 2 is presented as a methods figure. This is a critical component of the results as the authors have quantified blink duration systematically. I would place this section in the results, as a description of not only how blinks are detected, but how much varaibility there was across subjects.

6) Statistical issues. The authors find that females between 22 and 58 blink more than males, and yet there appears to be large individual variability. What percentage of that variability can be attributed to sex?

In a related fashion, how much variance in the blink probability is accounted for in the IPAST data when the subject is engaging in a pro- or anti- saccade task?
